# Targeting HOX/PBX dimers in cancer

**DOI:** 10.18632/oncotarget.15971

**Published:** 2017-03-07

**Authors:** Richard Morgan, Mohamed El-Tanani, Keith D. Hunter, Kevin J. Harrington, Hardev S. Pandha

**Affiliations:** ^1^ Institute of Cancer Therapeutics, Faculty of Life Sciences, University of Bradford, Bradford, UK; ^2^ Unit of Oral and Maxillofacial Pathology, School of Clinical Dentistry, University of Sheffield, Sheffield, UK; ^3^ Targeted Therapy Team, Chester Beatty Laboratories, The Institute of Cancer Research, London, UK; ^4^ Faculty of Health and Medical Sciences, University of Surrey, Guildford, UK

**Keywords:** HOX, PBX, HXR9, targeted therapy, biomarker

## Abstract

The *HOX* and *PBX* gene families encode transcription factors that have key roles in establishing the identity of cells and tissues in early development. Over the last 20 years it has become apparent that they are also dysregulated in a wide range of solid and haematological malignancies and have a predominantly pro-oncogenic function. A key mode of transcriptional regulation by HOX and PBX proteins is through their interaction as a heterodimer or larger complex that enhances their binding affinity and specificity for DNA, and there is growing evidence that this interaction is a potential therapeutic target in malignancies that include prostate, breast, renal, ovarian and lung cancer, melanoma, myeloma, and acute myeloid leukaemia. This review summarizes the roles of *HOX* and *PBX* genes in cancer and assesses the therapeutic potential of HOX/PBX dimer inhibition, including the availability of biomarkers for its application in precision medicine.

## HOX GENES IN CANCER

### The *HOX* genes

The *HOX* genes are a family of homeodomain-containing transcription factors that were originally identified due to their pivotal roles in early development [1]. These are reflected in a range of striking developmental changes in *HOX*-mutant animals, most notably *Drosophila*. One of the best known examples of this is the ectopic expression of the *Antennapedia* (*Antp*) gene in cells that would normally give rise to antennae; a near perfect pair of legs replaces the antennae of adult flies that develop from these embryos [2]. These observations revealed that *HOX* genes can determine the identity of cells and tissues, and hence also help regulate the proliferation, differentiation, and survival of these cells. The identification of additional mutants revealed a family of 8 *HOX* genes in the fly, which are expressed in an anterior to posterior pattern along the main embryonic axis and specify the identity of different embryonic structures [1].

An unusual feature of *HOX* gene organization is their existence in clusters within a single chromosome, and 2 further rounds of duplication in the course of vertebrate evolution have given rise to 4 chromosomal clusters in mammals, referred to as A, B, C, and D. The cluster names are also used to denote specific genes in conjunction with the position of the gene in the cluster, thus for example HOXD1 is the most 3′ member of the D cluster [3]. The genes within clusters share enhancer regions and this plays a significant role in the regulation of *HOX* gene expression, as do a number of microRNAs encoded within each cluster [4]. A further unusual feature of *HOX* regulation, which is in part enabled by enhancer sharing, is temporal and spatial collinearity during development, whereby each *HOX* gene in a cluster is expressed earlier in development and with a more anterior border of expression than its 5′ neighbour [1].

In total mammals have 39 *HOX* genes that play key roles in patterning both the main embryonic anterior to posterior axis at a very early stage of development and embryonic structures that develop later, for example the limbs and many of the organs [3]. The high level of sequence identity between *HOX* genes is reflected in a high level of functional redundancy during development [5], although there are also many examples of *HOX* genes playing specific roles in the embryo, for example during limb development where members of the HOXD cluster define specific structures [6].

Although *HOX* genes were originally characterized as developmental genes they also play a number of important roles in the adult, and indeed their original embryonic expression patterns are sometimes maintained, at least to a limited extent [7]. The most notable examples of *HOX* gene function in the adult include the maintenance of hematopoietic stem cells (HSCs) [8], the specification of different blood cell lineages [9], and regulation of tissue identity during implantation and the menstrual cycle [10]. In addition, and as discussed in detail below, the *HOX* genes become highly dysregulated (and often over expressed) in a wide range of both solid and haematological cancers.

### HOX cofactors

Although HOX proteins can bind to DNA through their homeodomain, this binding is relatively non-specific as it generally involves only a 4 base pair recognition sequence. Greater specificity is conferred by the binding of cofactors such Pre-B-cell Leukemia Homeobox (PBX) and Myeloid Ecotropic Viral Integration Site 1 Homolog (MEIS) proteins [11]. The latter bind to HOX proteins 9-13 [12], whilst PBX family members bind to HOX proteins 1-11 [13–15]. In addition to increasing the complexity of the DNA binding sequence, these cofactors also influence key transcriptional events such as the recruitment of RNA polymerase II or III, or transcriptional inhibitors such as HDAC. Hence, for example, HOXC6 can recruit RNA polymerase II through a TAAT site in the promoter of the *S100B* gene to promote its transcription in neuroblastoma cell lines [16], whilst HOXD3 needs to bind as a heterodimer with PBX1B to recruit RNA polymerase II and activate transcription of the *ITGB3* gene (encoding Integrin β3) during angiogenesis [17]. Conversely, HOXB6 and HOXD4 can bind to and inhibit the histone acetyl transferase CBP on the *TWIST1* enhancer, thereby repressing *TWIST1* transcription [18].

In addition to determining target gene specificity and the mode of transcriptional regulation, HOX cofactors also play a role in post-translational regulation through facilitating the entry of HOX proteins into the nucleus (considered in more detail below).

### The role of *HOX* genes in cancer

A potential role for *HOX* genes in cancer first became apparent from their frequent inclusion in chimeric, oncogenic gene fusions that drive the formation of haematological malignancies [9]. It has subsequently become apparent that *HOX* genes are profoundly dysregulated in a wide range of both solid and haematological malignancies, most frequently showing very high levels of over expression. There is now a vast amount of data available on *HOX* gene expression in different malignancies, and it is not within the scope of this review to detail this. The reader is instead referred to a number of recent specialist reviews that examine the expression and possible function of *HOX* genes in specific cancer types such as melanoma [19], and head and neck [4], prostate [20], breast [21], ovarian [22], and pancreatic cancer [23]. Despite the very high degree of *HOX* dysregulation in cancer, relatively few *HOX* genes have been shown to act as oncogenes in the strictest sense, i.e. that their forced expression in phenotypically normal cells is sufficient to cause a switch to a malignant phenotype, although an important example is *HOXA9*, which can immortalize normal bone marrow cells in mice when overexpressed [24]. Current evidence broadly indicates that other *HOX* genes have a general supportive role in malignancy, both at the cellular level (for example in promoting proliferation and blocking apoptosis) [25], and at the tumour level, where they have been shown to variously induce angiogenesis [26], drive metastasis [27], and facilitate drug [21, 28, 29] and radiation resistance [30]. These pro-oncogenic roles are reflected in numerous reports of elevated *HOX* expression being associated with a poor clinical outcome and prognosis (considered in more detail below).

In addition to a pro-oncogenic role, a number of *HOX* genes also act as tumour suppressors. Examples include *HOXA4*, the expression of which can block the spread of ovarian cancer cells [31], HOXA5 that has been shown to stabilize the P53 protein in breast cancer cells [32] and promote an epithelial phenotype [33], and *HOXC8* which is inversely related to progression in ovarian cancer [31]. Intriguingly it seems that the regulation of HOX tumour suppressor targets may involve HOX binding to the promoter or enhancer region without PBX, as the identified binding sites are often for HOX only [33, 34], or the HOX protein might have an additional function independent of that as a transcription factor [35].

### *HOX* genes as biomarkers

The differential expression of *HOX* genes in cancer make the products of these genes potential biomarkers for both diagnosis and prognosis, and for precision medicine applications such as predicting treatment response (reviewed by Morgan and El-Tanani, 2016 [36]). One of the best characterized examples is Engrailed-2 (*EN2*), a gene that is very closely related to the *HOX* genes but which is not located within the 4 main chromosomal clusters described above [37]. EN2 is overexpressed in a number of different malignancies, most notably prostate and bladder cancer, and urinary EN2 protein has been shown to be a potential diagnostic marker of both of these diseases [38, 39]. In addition, *HOXC6* RNA has been shown to be a potential diagnostic marker for prostate cancer as part of a multi gene panel [40]. *HOX* gene expression at the RNA level has also been shown to have prognostic significance in acute myeloid leukaemia (AML) [41], and in several solid malignancies including mesothelioma (HOXB4) [42], breast cancer (HOXB7) [43], ovarian cancer [44], oral squamous cell carcinoma (HOXD13) [45], thyroid cancer (HOXC10) [46], clear cell renal cell carcinoma (HOXC11) [47], gastric cancer (HOXC6) [48], and bladder cancer (HOXB13) [49]. Discreet patterns of *HOX* gene expression apparently exist within different types of cancer, indicating that they could be used to distinguish between cancer types when the primary tumour type is unknown, for example in circulating tumour cells (CTCs) [36].

## PBX GENES IN CANCER

### The *PBX* genes

*PBX* genes are homologues of the *Drosophila* extradenticle gene (*Exd*) and 4 are encoded in the human genome (PBX1-4). Like the *HOX* genes they encode homeodomain-containing transcription factors, but do not exist in chromosomal clusters. In addition to the homeodomain, PBX proteins contain other highly conserved regions, one of which is required for binding to a number of closely related transcription factors, MEIS and PREP [11]. PBX proteins also include 2 nuclear localization signals (NLSs) in the homeodomain region and a distinct nuclear export sequence (NES) [50]. The extent of nuclear localization of PBX proteins is determined by the balance between import and export pathways mediated by the NLSs and NES, respectively [51]. Unusually, the NES of PBX does not bind to exportin to directly promote nuclear exportation, but instead blocks access to the NLSs through binding to the homeodomain [52].

PBX proteins form strong complexes with HOX1-11 proteins in the presence of a HOX/PBX DNA binding consensus [53–55]. X-ray crystallography data for HOXB1-PBX1 and HOXB9-PBX1 complexes on DNA have revealed that each of the homeodomains binds to one half of an octameric consensus sequence, and that this interaction is stabilized by a HOX/PBX interaction mediated by a conserved hexapeptide sequenced in HOX proteins [15, 56–58]. Full length PBX1 alone cannot activate transcription, while a portion of it (amino acids 39-232) can specifically block transcriptional activation by the SP1 transcription factor [59, 60]. However, when complexed with HOXB1, PBX1 switches from a transcriptional repressor to a transcriptional activator [61].

### The role of *PBX* genes in cancer

Although less studied than the *HOX* genes in the context of cancer, the *PBX* gene family is known to have a number of oncogenic functions. Best characterised of these is as a chimeric fusion partner in various leukaemias and lymphomas, for example *PBX1* and *E2A* in pre-B cell acute lymphoblastic leukaemia [62]. It has also been shown that the upregulation of *PBX3* together with *MEIS1* is necessary to drive leukemogenesis efficiently in mouse models; PBX3 protein is required both to stabilize MEIS1 and induce transcription of the *MEIS1* gene [63]. Correspondingly, a 4-gene signature consisting of *PBX3*, *HOXA7*, *HOXA9*, and *HOXA11* has been shown to be an independent predictor of poor survival in patients with cytogenetically abnormal AML (CA-AML), and that *PBX3* (but not *PBX1* or *PBX2*) is frequently co-expressed with *HOXA9* in various subtypes of CA-AML, particularly MLL-rearranged AML, and may thus be a potential pathologic cofactor of HOXA9. This is further supported by the finding that knock-down of *PBX3* prevents it from exerting a synergistic effect with *HOXA9* in promoting leukemogenesis [64].

The *PBX* genes are also over expressed in a number of solid tumours. These include colorectal cancer (CRC), in which *PBX3* expression was shown to be correlated with invasive potential *in vitro*, and significantly associated with lymph node invasion, distant metastasis, advanced TNM stage and poor overall survival of patients. Furthermore, the forced expression of *PBX3* in cells with a low metastatic potential was shown to promote migration and invasion, at least in part through the upregulation of phosphorylated extracellular signal-regulated kinase (ERK)1/2 [65]. *PBX3* has also been shown to be upregulated in gastric cancer cells, and that this was associated with greater invasion depth, and advanced clinical stage and tumour grade. The overexpression of *PBX3* in gastric cancer cell lines with low endogenous *PBX3* expression accelerated cell proliferation and increased colony formation and cell-invading ability [66]. Similarly, increased levels of *PBX3* expression have been reported in prostate tumours and this was found to have prognostic significance in this cancer. *PBX3* expression is promoted by androgen signalling through a pathway that is negatively regulated in part by the Lethal-7 family of microRNAs (let-7), which are in turn downstream targets of the *Myc* protoncogene [67]. Although the majority of reports implicate *PBX3* as the most oncogenic *PBX* gene, a recent study demonstrated that increased *PBX1* expression in ovarian cancer was associated with shorter post-chemotherapy survival and increased resistance to platinum-based drugs through the maintenance of a stem cell-like phenotype [68].

## TARGETING HOX/PBX DIMERS

### Strategies for interfering with HOX/PBX dimer formation: HXR9 and other peptides

The key roles that HOX and PBX proteins play in cancer indicate that they are potential therapeutic targets. However, the high level of functional redundancy amongst HOX proteins and the general difficulty in producing effective small molecule inhibitors against transcription factors have proved significant barriers to this approach. As an alternative, it was proposed that the interaction between HOX and PBX proteins could be targeted, as this is mediated by a highly conserved hexapeptide sequence in HOX proteins and a hydrophobic binding pocket within PBX. Although a small molecule inhibitor of this interaction has previously been described, its Kd was in the micromolar range and it does not seem to have been adopted for experimental or clinical use [69]. To date, a more useful set of inhibitors have proved to be peptides that employ the hexapeptide sequence to act as a competitive antagonist of HOX/PBX binding. Several peptides have been described, but the one most frequently used is HXR9, an 18 amino acid peptide containing the hexapeptide sequence together with 9 arginine residues that promote cellular uptake by endocytosis. HXR9 was originally shown to be cytotoxic to melanoma cell lines and primary melanoma cells and was reported to reduce the growth of B16F10 murine melanoma tumours in an orthotropic model [25]. Subsequently, HXR9 was shown to inhibit the growth of a range of tumour types in mouse xenograft models, including non-small cell lung [70], breast [34], ovarian [71], and prostate cancer [72], and mesothelioma [42], melanoma [73], and meningioma [74] (Table 1).

**Table 1:** Previous *in vivo* studies of HOX/PBX inhibition using HXR9

Cancer (cell line)	Delivery (study duration)	Max % inhibition of tumour growth	Biomarkers investigated	Reference
Prostate (LNCaP)	100 mg/kg IT single dose when tumour volume > 100 mm^3^ (52 days)	530	*cFos* - tumour response	[72]
Breast (SKBR3)	20 mg/kg IT on weeks 1 and 2 (50 days)	340	*cFos* - tumour response Mean *HOXB1-9* expression - sensitivity	[34]
NSCLC (A549)	100 mg/kg IT single dose when tumour volume > 100 mm^3^ (18 days) or 10 mg/kg IP twice weekly (18 days)	880 (IT) 160 (IP)	*cFos*, EGR*1*, *EGR4* - tumour response	[70]
Mesothelioma (MSTO-211H)	25 mg/kg IP every 4 days, max 5 doses (37 days)	230	*cFos* - tumour response Ratio of mean “tumour suppressor” and “oncogenic” *HOX* expression - sensitivity	[42]
Melanoma (B16F10)	10 mg/kg IV twice weekly (28 days)	480	*cFos*, *DUSP1*, *ATF3* - tumour response	[25]
Melanoma (A375M)	100 mg/kg IT single dose when tumour volume > 100 mm^3^ (21 days)	440	*cFos* - tumour response	[73]
Ovarian (SKOV-3)	1 × 100 mg/kg IT (week 1) and then 10 mg/kg IT twice weekly, with or without cisplatin IP 3 mg/kg weekly (29 days)	140 (300 when combined with cisplatin)		[44]
Ovarian (SK-OV3)	1 × 100 mg/kg IV (week 1) and then 10 mg/kg IV weekly (32 days)	200	*cFos* - tumour response	[71]
Meningioma (IOMM-Lee)	30 mg/kg IV on days 7, 9, 13, 16, 19 (21 days)	170		[74]

Abbreviations: IT, intratumoral; IP, intraperitoneal; IV, intravenous; NSCLC, non-small cell lung cancer; “tumour response”, actual response of tumour to treatment; “sensitivity”, prediction of tumour sensitivity to treatment

**Figure 1:**
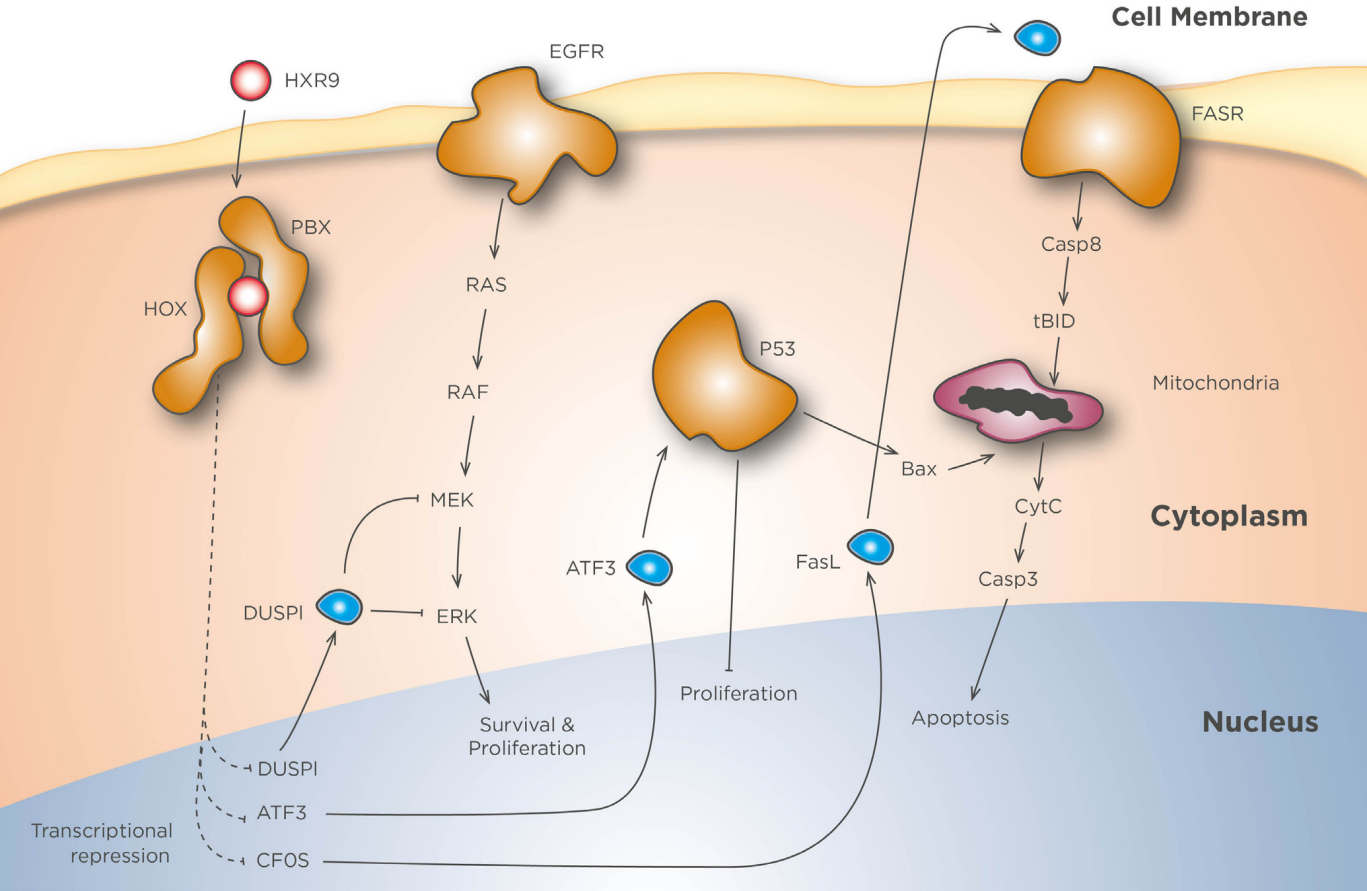
HXR9 mechanisms of action In the absence of inhibition HOX and PBX dimerize, enter the nucleus, and bind to HOX/PBX consensus sequences present in numerous target genes. These include *cFos, DUSP1,* and *ATF3*, all of which are inhibited by HOX/PBX dimers. HXR9 mimics the hexapeptide loop of HOX proteins that binds to a pocket in PBX, thereby acting as a competitive inhibitor of this interaction. HOX/PBX inhibition greatly increases *cFos* transcription, and the resulting protein can dimerize with Jun (not shown) to activate *FasL* transcription. FasL protein then binds to the Fas receptor (FasR) and activates the extrinsic apoptotic pathway. The transcription of *ATF3* and *DUSP1* is also enhanced by HXR9. ATF3 stabilizes p53, which in turn promotes mitochondria-mediated apoptosis and blocks proliferation, while DUSP1 dephosphorylates MEK and ERK, silencing Ras mediated signalling.

The mechanism by which HOX/PBX inhibition causes cell death remains to be fully elucidated (Figure 1). In most solid tumours cell death is mediated by apoptosis [25, 34, 42, 71, 72], although in a number of cancers, including some types of renal cancer, necrosis instead plays an important role [75]. However, both these events seem to be activated, at least in part, by a rapid increase in *cFos* expression, as preventing this using gene knock down strategies can achieve a partial rescue from HXR9-mediated cell killing [25]. The upregulation of *cFos* has been shown to cause apoptosis in a number of different cancers [76–83], in a mechanism that might involve activation of Fas ligand (FasL) transcription through the AP1 transcriptional activator consisting of the Fos/Jun heterodimer, which in turn promotes apoptosis through the FasL/Fas receptor pathway [76, 79–82]. High levels of *cFos* tumour expression are associated with longer survival in ovarian cancer patients [84], and, in addition to promoting apoptosis, it has also been shown to reduce the growth of ovarian xenograft tumours in mice through changes in cell adhesion [85]. *cFos* is an early response gene to many types of cellular stress, although there is now evidence to suggest that HOX/PBX dimers repress *cFos* expression both directly, through HOX/PBX binding sites in its promoter, and indirectly through HOX/PBX mediated regulation of a *cFos*-targeting microRNA [73].

### Predicting sensitivity to HOX/PBX inhibition

As with other types of targeted therapy, companion diagnostics are becoming increasingly important for patient stratification in clinical trials as well as personalized therapies. The identification of markers that can predict which tumours will be sensitive to HOX/PBX inhibition is an important preclinical goal, although to date there is relatively little evidence for a single or even multiplex marker that can fill this role outside of limited, cancer-specific settings. In a study on breast cancer, the sensitivity of breast cancer cell lines to killing by HXR9 was shown to be strongly associated with the combined expression of *HOX* genes *HOXB1* through to *HOXB9* [34]. However, this relationship has not been reported in other cancer types, and indeed it was not found amongst cell lines derived from mesothelioma, where instead there was an apparent relationship between HXR9-mediated cell killing and the ratio of expression in *HOX* genes with reported pro-oncogenic functions to those with tumour-suppressor functions [42]. Neither of these markers has been validated in primary tumours, although in breast cancer [34] and mesothelioma [42] a subgroup of primary tumours was identified that showed a very high level of the combined HOXB1-9 expression and a high HOX oncogenic to tumour suppressor ratio, respectively. Furthermore, in bone marrow samples from primary AML patients, high levels of HOXA gene and PBX3 expression were found to be associated with greater sensitivity to HXR9 [64].

The lack of a robust and generalized marker of tumour sensitivity to HOX/PBX inhibition might be related to the difficulty in quantifying these dimers in cells and tissues, as opposed to measuring HOX gene expression at the RNA or protein level, which is at best an indirect measure of this. Work is continuing to develop a quantitative assay for HOX/PBX dimers, and it is hoped that this will in turn lead to a more accurate test for tumour sensitivity to HOX/PBX inhibition.

### Biomarkers of response to HOX/PBX inhibition

Surrogate markers of tumour response to treatment, which allow an earlier assessment of the efficacy of treatment than conventional clinical endpoints (for example survival and disease free survival) are of growing importance both in clinical trials and in precision medicine. The regulatory function of HOX/PBX dimers means that there are a number of immediate transcriptional targets that change in response to HOX/PBX inhibition. These include *cFos*, as described above, together with a number of other targets that might act as a readout of the efficacy of inhibitors such as HXR9 [25]. The best characterised of these are dual specificity phosphatase 1 (DUSP1) and activating transcription factor 3 (ATF3). DUSP1 can dephosphorylate serine, threonine and tyrosine residues in a wide range of substrates, and block signalling through the mitogen activated protein kinase pathway, thereby preventing cellular proliferation [86], and decreased DUSP1 expression is associated with higher histological grades in multiple tumour types [87]. ATF3 also has a potential role in tumour suppression as it can stabilize p53 protein and prevent its ubiquination, induce apoptosis, and promote cell cycle arrest [88–90].

## FUTURE DIRECTIONS

There is now considerable *in vitro* and *in vivo* data supporting the therapeutic potential of inhibiting HOX/PBX dimer formation in cancer. To date, however, the only effective inhibitors of HOX/PBX binding are the HXR9 peptide and its derivatives. Peptide-based therapeutics are already used in cancer therapy though [91], including Carfilzomib for multiple myeloma [92], and indeed a variant of HXR9 for intratumoral injection is expected to enter clinical trials during 2018. Developing a small molecule inhibitor of HOX/PBX binding remains a significant challenge, and the only previously reported small molecule inhibitors of this interaction are unlikely to have any therapeutic application as the minimum reported Kd for PBX binding was 65 μM [69]. Development of a small molecule inhibitor therefor remains an important clinical goal.

## CONCLUSIONS

Since the publication of the initial study in 2007 it has become increasingly apparent that the HOX/PBX dimer is a potential therapeutic target in both solid and haematological malignancies. Clinical exploitation of this target is aided by the co-development of markers that predict the sensitivity of tumours to HOX/PBX inhibitors, and which allow the initial response to treatment to be monitored. The outcome of clinical trials for peptide and small molecule inhibitors of HOX/PBX are eagerly awaited.

## Abbreviations

AML: acute myeloid leukemia
ATF3: activating transcription factor 3
CA-AML: cytogenetically abnormal AML
CTC: circulating tumour cell
CRC: colorectal cancer
DUSP1: dual specificity phosphatase 1
EN2: Engrailed-2
FasL: Fas ligand
HSC: hematopoietic stem cell
MEIS: Myeloid Ecotropic Viral Integration Site 1 Homolog
NES: nuclear export sequence
NLS: nuclear localization signal
PBX: Pre-B-cell Leukemia Homeobox
